# A practitioner's perspective on interpreting QS Citations per Faculty

**DOI:** 10.3389/frma.2026.1808162

**Published:** 2026-06-19

**Authors:** Junjie Shen

**Affiliations:** Library Research Services, University of Bath, Bath, United Kingdom

**Keywords:** bibliometrics, Citations per Faculty, clarity, open science, QS, research assessment, university rankings

## 1 Introduction

University rankings have become powerful tools for institutional strategy and national research policy (Kochetkov, 2024). Within the QS World University Rankings, the Citations per Faculty (CPF) indicator accounts for 20% of the overall ranking score (QS Quacquarelli Symonds, 2025a). CPF is often treated as a proxy for research intensity, and its interpretation depends on a multi-stage construction process.

QS extracts citation data for CPF from Scopus early each calendar year. After this cut-off, institutions often examine their own Scopus records and form preliminary expectations about citation performance, before final ranking results are released later in the year. However, bibliometric normalization and rule-based adjustments can obscure the route from raw data to the final published score.

This article offers a practitioner-informed interpretation of the CPF pipeline. It treats QS as an illustrative case and argues that greater clarity in indicator design can support more transparent verification and use of citation-based metrics.

## 2 How CPF is constructed

**Figure 1:**
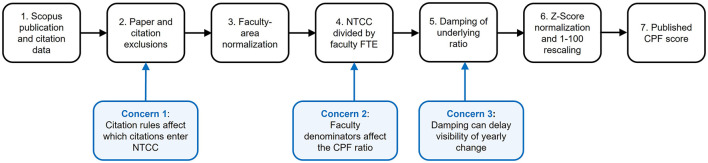
Simplified QS Citations per Faculty (CPF) construction pipeline. The annotations indicate where the three analytical concerns discussed in the paper enter the CPF pipeline.

QS presents CPF as a measure of citation volume relative to institutional faculty size. In simplified terms, the indicator starts from Scopus-derived publication and citation data, through paper and citation exclusions (QS Quacquarelli Symonds, 2025e), faculty-area normalization (QS Quacquarelli Symonds, 2022), division of the Normalized Total Citation Count (NTCC) by faculty full-time equivalent (FTE), damping—a mechanism that smooths large year-on-year changes by carrying forward only a portion of any improvement above a set threshold (QS Quacquarelli Symonds, 2025b), and final rescaling to a published score (QS Quacquarelli Symonds, 2024b) (Figure 1). This process means that CPF is a constructed indicator shaped by design choices that are not always visible to institutions.

The following sections examine three analytical concerns in this pipeline: the citation rules (Section 3.1), the faculty denominators (Section 3.2), and the damping mechanism (Section 3.3). For each concern, a hypothetical example sketches the effect of the relevant design choice on the citation input, underlying ratio, or year-on-year movement that a university might expect from its raw bibliometric and staffing data. The examples that follow illustrate plausible mechanisms; they do not establish the frequency or magnitude of these effects in actual QS calculations.

## 3 Three analytical concerns in the CPF pipeline

### 3.1 Citation rules

The first analytical concern relates to two rules governing which citations enter the NTCC: (1) the treatment of multidisciplinary publications classified under All Science Journal Classification (ASJC) code 1,000, and (2) the application of affiliation caps. QS documentation states that multidisciplinary publications classified under ASJC code 1,000 are reclassified based on the citation profile of the paper itself—that is, the subject codes most commonly found among papers it cites (QS Quacquarelli Symonds, 2025d). QS also applies affiliation caps to remove papers with unusually high numbers of affiliations (QS Quacquarelli Symonds, 2025e).

The two rules affect the citation input in different ways. For ASJC 1,000 publications, a multidisciplinary paper is counted only if it is successfully reassigned to a more specific subject area. A multidisciplinary paper may fail to be reassigned if its reference list does not contain a dominant ASJC subject area (for example, because the cited sources are too few or too dispersed across subject areas). In such cases, the paper remains in the ASJC 1,000 category and is excluded. This echoes a documented challenge in ranking methodologies: journal-based subject classifications may not always align with the actual research areas of individual publications, and such misalignment can affect ranking outcomes (Alshraideh and Abdelgawad, 2025).

For affiliation caps, a paper may be removed if the number of affiliations exceeds the relevant subject cap. Affiliation caps, therefore, limit the impact of very large collaborative papers whose citation counts could have disproportionate effects on institutional metrics, a concern also raised in recent bibliometric research on questionable authorship and affiliation practices (Meho and Akl, 2025). Both rules filter the citation record before NTCC is calculated, and neither outcome is currently visible at the article level to submitting institutions.

A hypothetical example in Table 1 depicts the effect. An institution may identify four Scopus-indexed papers with 2,100 citations in total, but after ASJC 1,000 treatment and affiliation-cap filtering, only 900 citations may be carried forward for NTCC calculation. This indicates that article-level information on ASJC 1,000 reassignment and affiliation-cap exclusions would make NTCC easier for institutions to verify and interpret.

**Table 1:** Hypothetical effect of ASJC 1,000 treatment and affiliation caps on citation input before NTCC calculation.

Paper	Raw citations	Treatment before NTCC	Citations carried forward
A	600	ASJC 1,000; reassigned to a QS subject area	600
B	400	ASJC 1,000; not reassigned	0
C	800	Exceeds affiliation cap	0
D	300	Included after filters	300
Total	2,100	—	900

The figures are illustrative and do not refer to any institution or actual QS calculation. “Not reassigned” refers to a scenario where an ASJC 1,000 publication cannot be mapped to a more specific QS subject area through the reassignment process.

### 3.2 Faculty denominators

The second analytical concern is the FTE denominator used in the CPF ratio. QS defines faculty staff as academic staff responsible for teaching, research, or both. It excludes support staff, PhD students who teach, hospital residents without teaching or research duties, visiting faculty, offshore staff, and inactive honorary or retired staff. QS also notes that research-only staff should be academically involved in research and likely to publish outputs (QS Quacquarelli Symonds, 2025c).

In practice, institutions may interpret these boundaries differently. One university may include research-only staff such as postdoctoral researchers and research fellows, while another university may exclude some postdoctoral or early-career researchers on the grounds that they support, rather than independently lead, research outputs. Similar variation may apply to clinical researchers, honorary appointments, and externally funded researchers. These differences matter because CPF is a ratio: holding NTCC constant, a lower faculty FTE denominator produces a higher CPF value. There are broader concerns that academic staff FTE is often insufficiently specified in ranking frameworks, raising questions about how productivity is assessed (Kochetkov, 2024). Table 2 depicts how different interpretations of faculty definitions can change the CPF ratio. To keep the example focused, it assumes that part-time staff have already been converted into FTE consistently. The variation shown therefore comes only from how research-only staff are interpreted under the faculty definition. Variation could also arise from the conversion of part-time contracts into FTE, because QS provides both a suggested formula, FTE = full-time count + (part-time count/3), and an average calculation based on actual commitment hours (QS Quacquarelli Symonds, 2025c).

**Table 2:** Hypothetical effect of faculty FTE interpretation on CPF ratio.

Staff component	Wider interpretation FTE	Narrower interpretation FTE
Teaching and research staff	700	700
Teaching-only staff	100	100
Research-only staff	200	100
Total faculty	1,000	900
NTCC	90,000	90,000
CPF ratio (NTCC/FTE)	90	100

The figures are illustrative and do not refer to any institution or actual QS calculation. The example assumes the same staff population and NTCC in both scenarios; the difference is whether all or some research-only staff are judged to meet the faculty definition.

National reporting systems also differ. In the United Kingdom, the Higher Education Statistics Agency (HESA) provides a public reference point for staff data, and QS applies filters to align HESA staff data with its own faculty definitions (QS Quacquarelli Symonds, 2024a). In many other countries, comparable public staffing data may be unavailable, leaving faculty numbers dependent purely on institutional submission and making external verification difficult. As a result, ranking indicators may influence institutional decisions about staff classification and reporting. This reflects a well-documented dynamic in rankings research: when metrics carry high stakes, they tend to shape the institutional behaviors they were designed to measure (Morgan-Thomas et al., 2024; Hladchenko, 2025).

### 3.3 Damping mechanism

The third analytical concern is the damping mechanism applied to the underlying CPF ratio. QS states that damping is used to smooth large year-on-year swings in the underlying data, and that for non-reputation indicators, it is the underlying ratios or indices that are damped. For CPF, QS lists a relative damping threshold of 0.1, a recovery increment of 20%, and a damping limit of 2 (QS Quacquarelli Symonds, 2025b). In practice, when the underlying CPF ratio rises above its previous damped value, only a portion of that increase feeds through to the published score. Table 3 illustrates the effect using hypothetical figures. Suppose an institution's previous damped CPF ratio is 100, and its underlying CPF ratio rises to 150 in Year 1. The relative threshold of 0.1 allows the first 10% of the previous damped value to be carried forward in full, bringing the value to 110. The remaining excess is 40, and 20% of this excess is recovered, giving 110 + 8 = 118. The damping limit of 2 confirms that damping applies in this example, since both the previous damped value and the current underlying CPF ratio are well above 2; the calculation proceeds using the relative threshold and recovery increment. The same rule is applied in subsequent years using the previous year's damped value as the new baseline. To isolate the damping effect, the example assumes that the underlying ratio remains at 150 from Year 1 to Year 4. The damped value then approaches the underlying ratio gradually over the years.

**Table 3:** Hypothetical effect of CPF damping on a year-on-year improvement.

Year	Underlying CPF ratio	Previous damped value	Threshold value	Damped value carried forward	Visible improvement (%)
Year 0	—	—	—	100.0	—
Year 1	150.0	100.0	110.0	118.0	36.0
Year 2	150.0	118.0	129.8	133.8	67.7
Year 3	150.0	133.8	147.2	147.8	95.6
Year 4	150.0	147.8	162.6	150.0	100.0

The figures are illustrative and do not refer to any institution or actual QS calculation. Visible improvement is calculated as the increase in the damped value from the Year 0 baseline, divided by the total underlying increase from 100 to 150.

It should be noted that the damped underlying CPF ratio in Table 3 is not the final published score. The published CPF score is derived from the damped ratio through a further z-score normalization step, which means the magnitude of the visible improvement in the published score will also depend on the distribution of scores across all ranked institutions (QS Quacquarelli Symonds, 2024b).

From an institutional perspective, the damping mechanism means that published CPF scores might not be a real-time reflection of citation performance. A genuine improvement in citation activity may take several ranking cycles to appear fully, making year-on-year score movements an unreliable signal for internal evaluation.

This time-lag effect also has implications for transparency in research evaluation. Frameworks such as the UNESCO Recommendation on Open Science (UNESCO, 2021) and the Agreement on Reforming Research Assessment (Arentoft et al., 2022) emphasize openness and transparency in research assessment systems. In this context, clearer communication about the way damping operates would help institutions better understand the temporal relationship between bibliometric data, damped underlying ratios, and published ranking outcomes.

## 4 Conclusion

This article argues that the QS CPF should be interpreted as a constructed indicator rather than a direct citation-per-academic measure. The worked examples illustrate three analytical concerns: citation rules that may cause institutional checks to differ from the citation count used in CPF; faculty FTE definitions that may alter the denominator even when the citation numerator is unchanged; and damping that may suppress year-on-year changes in the underlying ratio for multiple cycles.

Given CPF's 20% weighting in the QS World University Rankings, these design choices have practical implications for how institutions interpret the indicator, although their effects will depend on each institution's bibliometric profile, staffing data, and position within the wider ranking distribution. The contribution of this article is diagnostic and illustrative: it identifies points in the CPF pipeline where verification and interpretation may become difficult, rather than empirically validating or critiquing the CPF indicator as a whole. The need for clearer methodological visibility is part of ongoing debates about ranking transparency and responsible use (Gadd, 2021). More detailed article-level information on citation exclusions and reassignment, more explicit guidance on faculty FTE interpretation, and worked examples of damping would reduce the verification gap and support more precise interpretation of CPF results.
